# Infection of polarized bovine respiratory epithelial cells by bovine viral diarrhea virus (BVDV)

**DOI:** 10.1080/21505594.2020.1854539

**Published:** 2021-01-04

**Authors:** Ang Su, Yuguang Fu, Jochen Meens, Wei Yang, Fandan Meng, Georg Herrler, Paul Becher

**Affiliations:** a Department of Infectious Diseases, Institute of Virology, University of Veterinary Medicine Hannover, Foundation, Hannover, Germany; b State Key Laboratory of Veterinary Etiological Biology, Lanzhou Veterinary Research Institute, Chinese Academy of Agricultural Sciences, Lanzhou, China; c Institute of Microbiology, University of Veterinary Medicine Hannover, Foundation, Hannover, Germany; d College of Veterinary Medicine, Northeast Agricultural University, Harbin, China; e State Key Laboratory of Veterinary Biotechnology, Harbin Veterinary Research Institute, Chinese Academy of Agricultural Sciences, Harbin, China

**Keywords:** Bovine viral diarrhea virus (BVDV), pestivirus, cattle, bovine respiratory disease complex, epithelial barrier, bovine polarized respiratory epithelial cells, entry and release of BVDV, cd46

## Abstract

Bovine viral diarrhea virus (BVDV) is affecting cattle populations all over the world causing acute disease, immunosuppressive effects, respiratory diseases, gastrointestinal, and reproductive failure in cattle. The virus is taken up via the oronasal route and infection of epithelial and immune cells contributes to the dissemination of the virus throughout the body. However, it is not known how the virus gets across the barrier of epithelial cells encountered in the airways. Here, we analyzed the infection of polarized primary bovine airway epithelial cells (BAEC). Infection of BAEC by a non-cytopathogenic BVDV was possible via both the apical and the basolateral plasma membrane, but the infection was most efficient when the virus was applied to the basolateral plasma membrane. Irrespective of the site of infection, BVDV was efficiently released to the apical site, while only minor amounts of virus were detected in the basal medium. This indicates that the respiratory epithelium can release large amounts of BVDV to the environment and susceptible animals via respiratory fluids and aerosols, but BVDV cannot cross the airway epithelial cells to infect subepithelial cells and establish systemic infection. Further experiments showed that the receptor, bovine CD46, for BVDV is expressed predominantly on the apical membrane domain of the polarized epithelial cells. In a CD46 blocking experiment, the addition of an antibody directed against CD46 almost completely inhibited apical infection, whereas basolateral infection was not affected. While CD46 serves as a receptor for apical infection of BAEC by BVDV, the receptor for basolateral infection remains to be elucidated.

## Introduction

Along with classical swine fever virus (CSFV), border disease virus (BDV), atypical porcine pestivirus (APPV), bovine viral diarrhea virus (BVDV) belongs to the genus *Pestivirus* within the family *Flaviviridae* [1,2]. Like other pestiviruses, BVDV is an enveloped, positive-strand RNA virus. Two species, *Pestivirus A* and *B*, also designated BVDV-1 and BVDV-2, can be distinguished. For BVDV-1, at least 21 different subgenotypes have been described, while BVDV-2 comprises four subgenotypes [3]. Depending on the capacity to induce a cytopathic effect in cell culture or not, two biotypes of BVDV, cytopathic (cp) and non-cytopathic (ncp) strains, are distinguished [4]. In general, infection of adult animals by ncp BVDV results in a systemic infection that may be associated with acute respiratory and gastrointestinal disease, immunosuppressive effects, and reproductive failure in cattle [5–10]. An ncp BVDV can cross the epithelial barrier of the placenta to induce infection of the fetus resulting in fetal death, abortion, or the birth of persistently infected (PI) animals [11–13]. PI animals shed large amounts of viruses for the rest of their life and are the most important sources of virus spread [11,14,15].

The viral envelope glycoprotein E2 is a crucial determinant of the cellular tropism of BVDV [16,17]. It mediates virus attachment by binding to the cellular receptor, membrane cofactor protein (CD46) which is the only known receptor identified so far for BVDV [18]. However, the available evidence suggests that CD46 is an attachment receptor that is not sufficient for virus entry [19,20]. Therefore, interaction with other proteins that act as co-receptors or alternative receptors may be necessary for virus entry [19,20]. The ncp BVDV can be transmitted in a wide range of body fluids, including nasal discharge, saliva, tears, urine, milk, semen, and fetal fluids [6,21,22]. Following exposure, the initial infection occurs within the oronasal mucosa and the tonsils [23–25]. After breaking through the epithelial barrier, the virus gets access to the regional lymph nodes for further dissemination by BVDV-sensitive lymphocytes and monocytes leading to systemic spread [26,27]. However, very little is known about the initial stage of infection, especially about the mechanism of how BVDV overcomes the epithelial barrier in different organs (airway, intestine, and placenta), resulting in viremia and systemic virus spread.

To prevent infection, the airway epithelial cells serve as a primary barrier acting as a physical barrier, a chemical barrier, and an immunological barrier to prevent the invasion of microorganisms [28,29]. The airway system is lined by polarized epithelial cells [30,31]. The plasma membrane of these cells is subdivided into apical and basolateral domains that are separated by tight junctions [32]. The apical membrane faces the external environment, whereas the basolateral membrane has contact with the internal tissues and blood vessels [33]. Special sorting events ensure that proteins and lipids specific for either of the two surface domains are transported to the correct target membrane [30,34]. These specific properties are crucial for the outcome of infections [35,36]. Whereas virus entry is dependent on the apical/basolateral distribution of viral receptors, virus release is determined by the localization of the viral membrane proteins and/or the matrix protein [37,38]. Polarized entry and release of viruses affect the course of the airway infection [39,40]. Respiratory viruses usually initiate infection of the airway epithelium by entering cells via the apical plasma membrane [41]. Subsequently, the spread of infectious particles can occur in two different ways. In localized infections, virus egress is confined to the apical surface of epithelial cells, i.e. entry and release occur via the same membrane domain. By contrast, in systemic infections, viruses usually enter apically and exit at the basolateral side [42,43].

We addressed the question of how BVDV gets across the airway barrier of polarized epithelial cells to induce systemic infection. By the establishment of a culture system of bovine airway epithelial cells, we are able to analyze the polarity of BVDV infection. Here, we present data on directional BVDV entry and exit pathways in polarized cells. In addition, we demonstrate the distribution of CD46 and investigate its role for BVDV entry in polarized cells.

## Material and methods

### Establishment of polarized bovine airway epithelial cells

Fresh lungs were collected from calves at a local slaughterhouse. Bovine primary bronchial epithelial cells (PBEC) were isolated as previously described [44] and were expanded in growth medium (BEGM). When the PBEC had reached confluence, 5 × 10^5^ cells were seeded on the apical compartment of Corning Transwell® polycarbonate membranes with 300 µl medium in the apical compartment and incubated for 24 h at 37°C in a humidified 5% CO_2_ atmosphere, while the basolateral compartment was filled with 600 µl medium. The transepithelial electrical resistance (TEER) was measured every day by using the Millicell® ERS-2 Voltohmmeter (Millipore) according to the manufacturer’s instructions. Only monolayers with TEER of more than 500 Ω were used for the infection experiments [45]. Moreover, FITC-labeled 70,000-molecular-weight (Da) dextran (Invitrogen) was added to the apical compartment to measure the integrity of the epithelial barrier. The medium was harvested from the basolateral compartment at different time points, and the fluorescence was determined with a spectrophotometer (Varian Cary Eclipse).

### Cells and viruses

Madin-Darby bovine kidney (MDBK) cells were obtained from the American Tissue Culture Collection, Rockville, Maryland, USA. The non-cytopathogenic BVDV-1 strain NCP7 was used throughout this study [46]. Cells were grown in Dulbecco’s modified Eagle’s medium supplemented with 10% horse serum. Before usage, the horse serum was analyzed by specific PCRs to ensure the absence of BVDV or *Mycoplasma*. In addition to the medium, the horse serum was inactivated at 50°C for 1 h. Every 3 months, the cells were tested by RT-PCR and immunofluorescence analysis to exclude potential contamination by BVDV.

### Virus infection

Prior to infection experiments, the filter-grown cells were washed with warm phosphate-buffered saline (PBS). The filters were inoculated with diluted virus inoculum from the apical or basal side at a multiplicity of infection (MOI) of 0.5 for 1 h. Control cells were mock-infected with PBS. The cell number per filter support was approximately 5 × 10^5^. After the unbound virus had been removed by washing, the infected cell layers were maintained with 150 µl of culture medium in the apical chamber and 600 µl in the basolateral chamber for 3 days at 37°C and 5% CO_2_. At different time points, volumes of 100 µl medium were collected from the apical chamber and basolateral chamber of each infected and uninfected filter and replaced by the same volumes of fresh medium, respectively. The harvested samples were titrated by endpoint titration on MDBK cells to assess the virus infectivity as previously described [47].

### CD46 blocking assay

To assess the importance of the receptor CD46 for BVDV infection on polarized airway epithelial cells, a CD46 blocking assay was performed. Prior to infection with BVDV NCP7, cells were treated with the monoclonal antibody (mAb) CA17 directed against CD46 [18]. The antibody was applied either only to the apical or to the basolateral or to both membrane domains. Cells were infected at an MOI of 0.25. After 1 h, the unbound virus was removed by six washes with PBS. Culture medium was added, 150 µl to the apical chamber and 600 µl to the basolateral chamber. To continue the blocking effect, the medium contained the CA17 mAb, as in the pretreatment. The infected cells were incubated at 37°C for 48 h for further analysis.

### Immunofluorescence microscopy

Cells were washed with PBS three times and fixed with 3% paraformaldehyde (PFA) for 20 min. PFA was removed and 0.1 M glycine was added for 5 min. Subsequently, the cells were permeabilized with 0.2% Triton X-100, washed three times with PBS and further blocked with 5% goat serum, and finally incubated with primary antibody and secondary antibody for 1 h each. After washing with PBS, the nuclei were incubated with DAPI (4′,6-diamidino-2-phenylindole), embedded in Prolong Gold Antifade Reagent (Life Technologies), and stored at 4°C for further analysis. The primary antibodies used in this study were as follows: anti-ZO-1 antibody (Life Technologies) for staining tight junctions, and anti-β-catenin antibody (Sigma) for staining a basolateral marker protein. It should be noted that the latter staining is not efficient and only detects cells that show a strong expression of ß-catenin. The monoclonal antibodies against BVDV NS3 (BVD/C16) and bovine CD46 (CA17) have been previously described [18]. All antibodies were diluted in 1% bovine serum albumin and incubated at RT for 1 h. Green fluorescence and red fluorescence (Alexa Fluor® 488 and 568) conjugated antibodies (1:1000, Life Technologies) were used as secondary antibodies. Samples were analyzed by using the Nikon Eclipse Ti-S microscope (Nikon) and TCS SP5 confocal laser scanning microscope (Leica). For analysis of images, NIS-Elements Viewer 4.20 software (Nikon), LAS AF Lite software (Leica), and ImageJ/Fuji software were used.

### Statistical analyses

All in vitro-experiments were conducted at least three times and data were subjected to statistical analysis using GraphPad Prism 8 software with Tukey multiple comparison test. Results were shown as means with standard deviations. A p-value of <0.05 was considered significant.

## Results

### Establishment of polarized bovine airway epithelial cells

**Figure 1.**
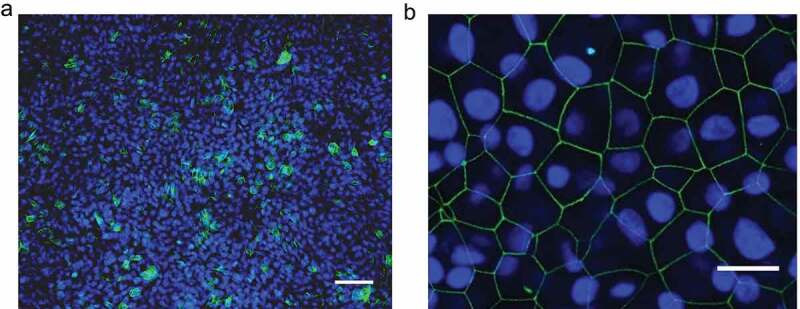
**Morphological examination of bovine polarized airway epithelial cells**. The bovine airway epithelial cells were seeded on transwell filter for 24 h. Prior to the infection by BVDV, the mock filter samples were evaluated by immunofluorescence analysis to examine the polarized status. (a) Staining of β-catenin as the basolateral protein marker expressed on the epithelial monolayer in green color. (b) The tight junction protein ZO-1 was visualized by immunofluorescence staining 24 h after seeding of cells. Scale bar, 50 µm (A), 20 µm (B)

**Figure 2.**
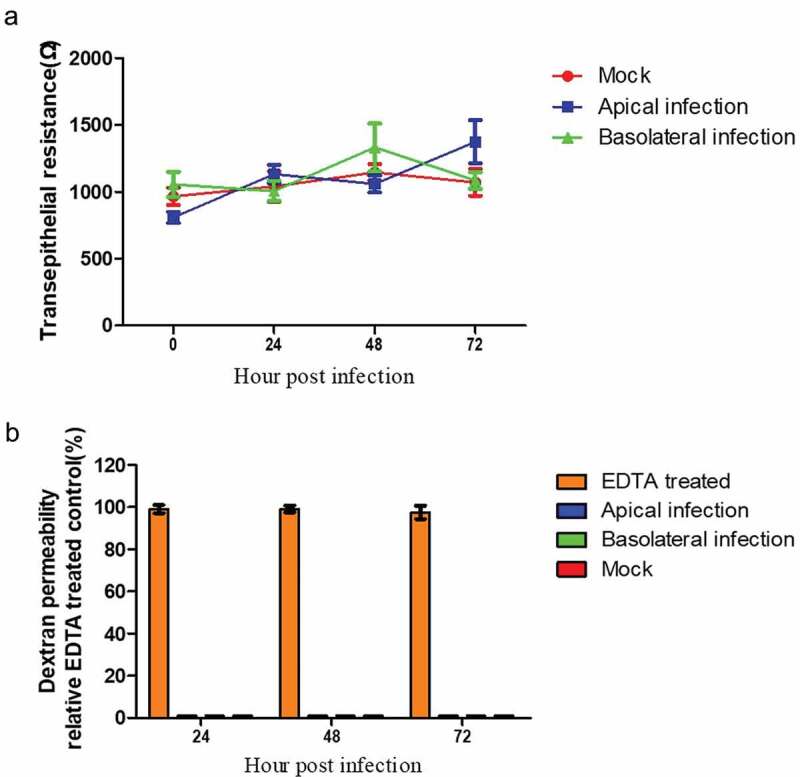
**Maintenance of the barrier function of virus-infected polarized epithelial cells**. In order to study whether or not the barrier function of polarized epithelial cells was disrupted by BVDV infection, two methods to assess the epithelial barrier function were applied: the transepithelial electrical resistance (TEER) assay (a) and the FITC-labeled 70,000-molecular-weight (Da) dextran assay (b). (A) The TEER was monitored in the presence or absence of the virus up to 72 h postinfection. Despite infection of the cells by BVDV, the TEER values didn’t significantly change in the observation period. (B) FITC-labeled 70,000-molecular-weight (Da) dextran was added to the apical compartment of infected and uninfected cells and subsequently, its presence in the basolateral compartment was examined by the FITC fluorescence in a photometer at the indicated time points. This analysis proved that the barrier integrity was sustained in the cell cultures

To establish a culture system for polarized bovine airway epithelial cells, primary calf bronchial epithelial cells were collected and cultured on transwell filters. At 24 h after seeding on the filter, the cell layers were analyzed by immunofluorescence microscopy to investigate whether they had adopted a polarized organization form. β-catenin, a basolateral marker protein, was expressed forming a ring along the borders of the cells Figure 1a. Moreover, the presence of tight junctions was demonstrated by the positive staining for ZO-1 Figure 1b. These results indicated that the monolayer consisted of polarized epithelial cells. This conclusion was confirmed by the measurement of the transepithelial electrical resistance (TEER) (Figure 2a, mock).

### Maintenance of the barrier function after BVD virus-infection

To determine whether BVDV infection affects the barrier function of polarized airway epithelial cells, the TEER was measured. During the whole infection period analyzed, the TEER values of polarized airway epithelial cells were not decreased but sustained at a stable level after infection by BVDV NCP7 for up to 72 h postinfection Figure 2a. The effect of BVDV infection on the barrier function of the epithelial cell layer was also analyzed by determining the permeability for large molecules. FITC-labeled 70,000-molecular-weight (Da) dextran was added to the apical compartment. As shown in Figure 2b, there was no fluorescence leakage detected in the basolateral filter compartment regardless of whether the cells were infected or not. These results indicate that tight junctions remain in a functional state during BVDV infection of polarized airway epithelial cells preventing the transcellular diffusion across the epithelial cell layer.

### Polarity of virus entry

**Figure 3.**
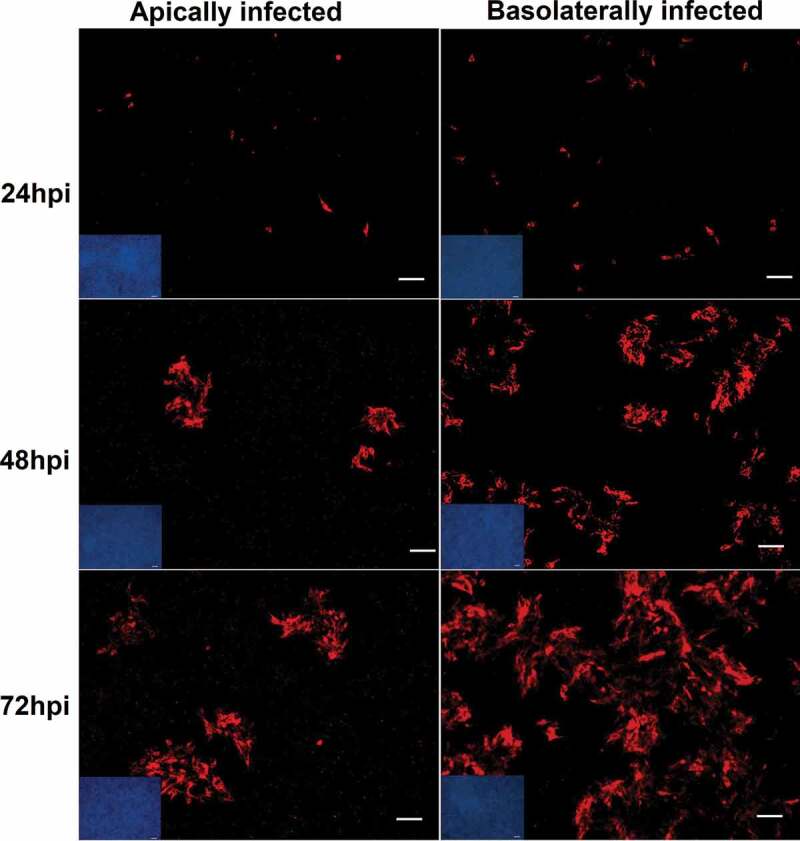
**Immunofluorescence analysis of bovine polarized epithelial cells at different time points after infection with BVDV NCP7 from apical (left) and basolateral (right) side**. Bovine polarized airway cells were infected apically and basolaterally by BVDV at an MOI of 0.5 and incubated for 72 h at 37°C. Expression of BVDV NS3 was visualized by immunofluorescence staining using monoclonal antibody BVD/C16 (red), the nuclei were stained by DAPI (blue). The insets show the nuclei staining by DAPI demonstrating the confluency of the cells. The virus can initiate infection from both apical domain and basolateral domain. However, infection from the basolateral compartment is characterized by a more efficient spread compared to infection from the apical side. Scale bars represent 25 µm. All experiments were performed at least three times

To analyze how BVDV enters polarized bovine airway epithelial cells, filter-grown cells were infected from either the apical or basal side by BVDV NCP7 at an MOI of 0.5. The filters were incubated for up to 72 h postinfection and cells were collected at different time points for immunofluorescence staining. As shown in Figure 3, BVDV-infected cells were found after both apical and basolateral infection. However, a significantly higher number of cells were infected after basolateral infection. These results indicate that BVDV can enter the airway epithelial barrier from both the apical and the basolateral side. However, infection via the basolateral plasma membrane is more efficient compared to infection via the apical membrane compartment.

### Replication kinetics of BVDV and virus release from polarized bovine airway epithelial cells

**Figure 4.**
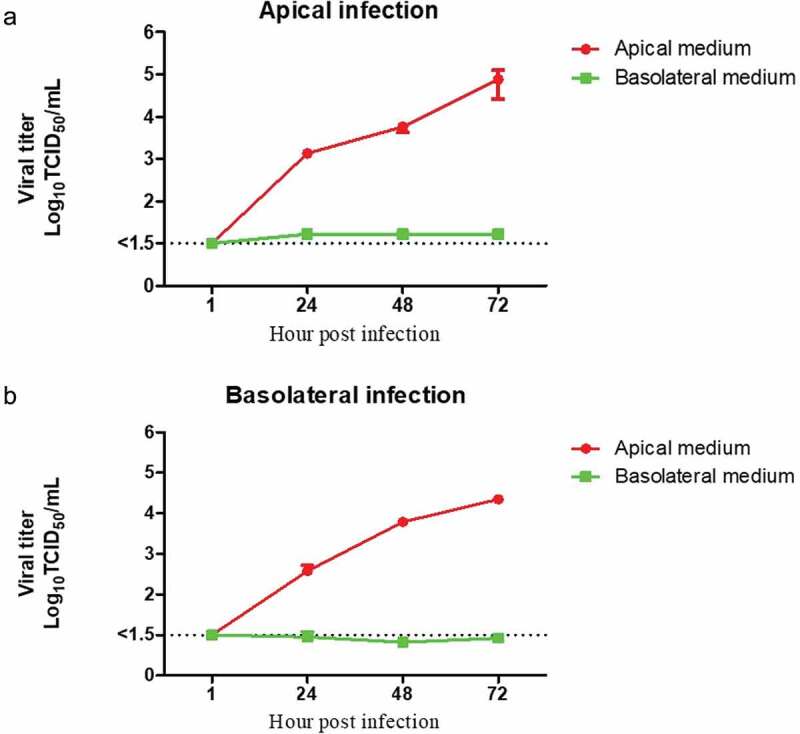
**Release of BVDV in bovine polarized airway epithelial cells**. To determine the viral replication kinetic and viral releasing pattern in bovine polarized airway epithelial cells after BVDV infection via the apical (a) and basolateral (b) side. The apical medium and basolateral medium from each infected filter were collected at the indicated time points after infection by BVDV NCP7. After infection from both sides (apical and basolateral), virus release is prone to the apical surface, whereas only very few viruses can be detected at the basolateral side. The results were shown as mean values ±SEM of nine polarized cell cultures from three independent donors. The dash lines indicate detection limits for the assays

To analyze the replication of BVDV in polarized airway epithelial cells, filter-grown cells were infected from either the apical or the basolateral side at an MOI of 0.5 and incubated for up to 72 h. Aliquots of the supernatants were collected at 24 hpi, 48 hpi, and 72 hpi from both the apical and the basolateral chamber and analyzed for the presence of infectious viruses. As shown in Figure 4(a,B) the course of infection after the apical and basolateral infection was similar. Infectious virus was detected 24 hpi and the amounts of viruses increased by 72 hpi. Infectious viruses were released most efficiently via the apical plasma membrane; in contrast, only very low amounts of viruses were detected in the basal medium Figure 4.

### The distribution of CD46 in polarized bovine airway epithelial cells

**Figure 5.**
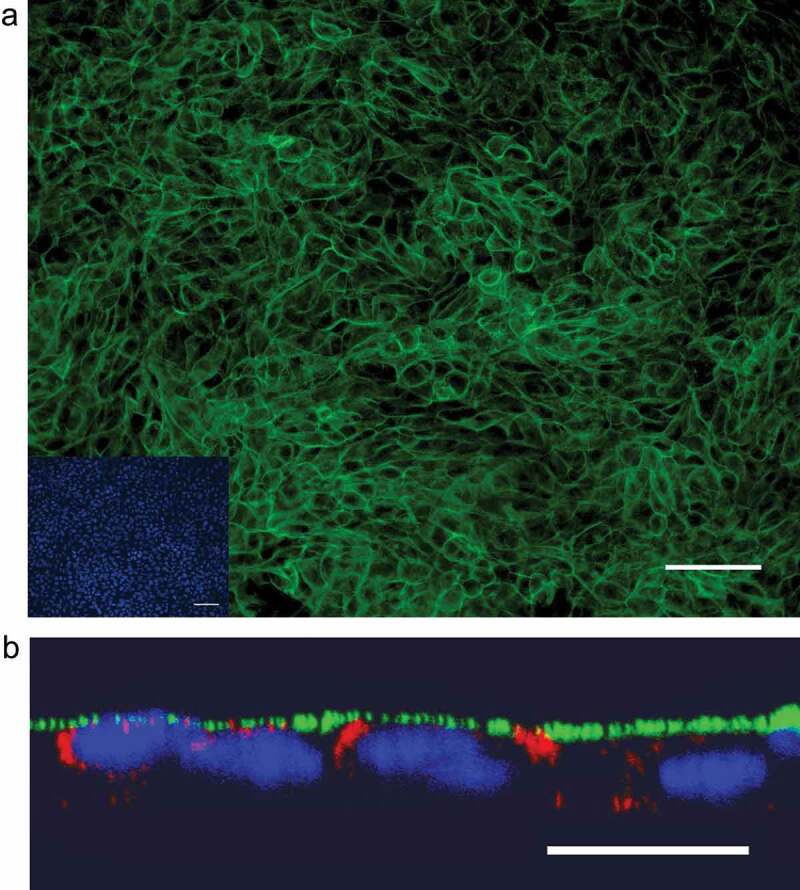
**Distribution pattern of CD46 on bovine polarized airway epithelial cells**. (a) Horizontal section. CD46 expression was detected by immunofluorescence analysis using monoclonal antibody CA17 (green). Nuclei were visualized by DAPI staining (blue). Scale bar: 50 µm. (b) Vertical section. Immunofluorescence staining of CD46 (green) and β-catenin (red). Scale bar: 10 µm

Membrane cofactor protein CD46 is a complement regulatory protein that has been identified as a receptor for BVDV in the initiation of infection. Therefore, we examined the distribution pattern of CD46 in polarized airway epithelial cells and compared it with that of BVDV-infected cells. Twenty-four hours after seeding of cells on transwell filters selective filter samples with TEER values of ≥500 Ω were collected for immunofluorescence analysis. Antibody CA17 directed against bovine CD46 was applied on the fixed cell layers to explore the expression of CD46. Also, β-catenin, a basolateral plasma membrane marker was included in the immunofluorescence analysis. Examination of horizontal sections demonstrated that CD46 was detectable on the surface of polarized epithelial monolayer Figure 5a. The analysis of vertical sections showed that CD46 was predominantly expressed on the apical plasma membrane of polarized epithelial cells. Distribution of CD46 can hardly be found at the basolateral plasma membrane Figure 5b. Furthermore, the infection of epithelial cells with BVDV did not affect the polarized location of CD46. Our results indicate that CD46 is an apical protein and that this property is maintained after infection by BVDV.

### Importance of CD46 during infection of polarized respiratory epithelial cells by BVDV

**Figure 6.**
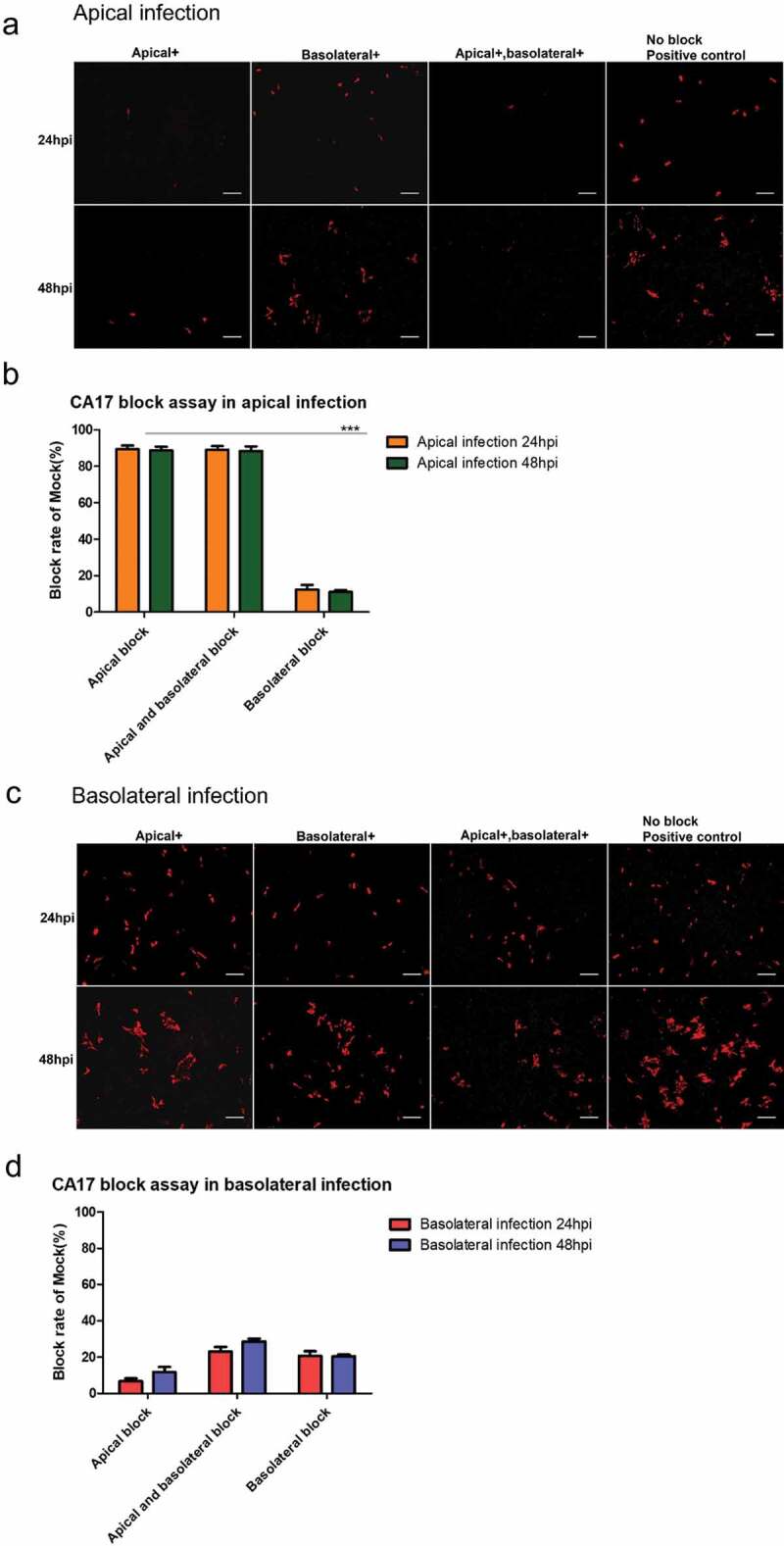
**CD46 blocking assay on polarized airway epithelial cells infected by BVDV**. Polarized airway cells were treated with anti-CD46 antibody prior to infection. Three blocking groups were analyzed in this experiment: “apical blocking group” (Apical+), “basolateral blocking group” (Basolateral+) and “apical and basolateral blocking group” (Apical+, Basolateral+). Each sample in those groups was infected by BVD NCP7 at an MOI 0.25 from apical side (a and b) and basolateral side (c and d), respectively. Panels A and C show the results of immunofluorescence staining of cells 24 h and 48 h after infection. (a) Apical infection. In apical infection scenario, BVDV infection was almost completely blocked by treatment of the apical medium with the anti-CD46 antibody (“apical blocking group” and “apical and basolateral blocking group”) compared to positive control group. In contrast, treatment of the basal medium with the anti-CD46 antibody (“basolateral group”) had no significant effect on apical BVDV infection. (b) Quantitative analysis of the blocking rate in apical infection condition. (c) Basolateral infection. BVDV can overcome the blocking effect to infect polarized cells at basal side though in the “apical and basal blocking” group an up to 30% blocking rate was observed. (d) Quantitative analysis of the blocking rate of infection after basolateral infection. For each blocking study, a total of 6 polarized samples from three independent donors were performed. Additionally, three fields per culture were evaluated as technical replicates. Scale bars, 50 µm

To get information on the role of CD46 during BVDV infection, a CD46 blocking assay was performed. Prior to virus infection, cells were incubated with antibody CA17 for 1 h. Then, BVDV was added at an MOI of 0.25. As CD46 was expressed predominantly on the apical surface, we differentiated between three different scenarios. The monoclonal antibody was applied to the apical or the basolateral compartment or both. At 24 and 48 hpi, cells were analyzed by immunofluorescence microscopy for the presence of infected cells. As shown in Figure 6, CA 17 was indeed able to inhibit BVDV infection; however, only when cells were infected from the apical side Figure 6 (A,B) and the blocking antibody was present in the apical compartment (samples “apical” and “apical + basolateral”). When basolateral infection Figure 6 (C,D) was analyzed, only a minor reduction in the number of infected cells was determined.

## Discussion

In viral infections that are restricted to the respiratory tract, virus entry and egress usually occur via the apical plasma membrane [36,37,39]. For the respective viruses, it is sufficient if the receptor for virus entry is present on the apical domain of the plasma membrane [48]. Viruses that establish systemic infections frequently use the airways only as a transit station from where they spread to other organs or tissues. For BVDV, it has been reported that intranasal infection is the most common route of infection and that the virus first replicates in the nasal mucosa and to high titers in the tonsil(s?), followed by spread to the regional lymph nodes and dissemination throughout the body [24–26]. Accordingly, if BVDV wants to invade and leave its host via infection of the airway epithelial cells, it would need a receptor at the apical surface for initiating infection and on the basolateral domain of the plasma membrane for the final infection before leaving the host. To study BVDV infection in the bovine respiratory tract, polarized non-differentiated airway epithelium cells were established. For all experiments, a non-cytopathic BVDV strain was used because this biotype is predominantly circulating in the field and sustains BVDV infections in the cattle population [3,14,22,49,50]. Furthermore, cytopathic BVDV strains which usually emerge from non-cytopathic BVDV by RNA recombination within persistently infected animals are less relevant for infections of the respiratory epithelium by BVDV. One important conclusion resulting from the present study is that BVDV can efficiently replicate in the airway epithelium after both apical and basolateral infection Figure 3, but is efficiently released only from the apical side Figure 4. Accordingly, the airway epithelium can serve as an entry site of infection and local replication for BVDV followed by efficient virus release to the apical compartment, thereby promoting spread to other animals via aerosols and droplets [2,15,51,52]. However, the lack of virus release to the basolateral side indicates that BVDV cannot cross the airway epithelium to directly infect subepithelial cells. As it is well known that BVDV can replicate to high titers in macrophages, dendritic cells, and lymphocytes, it appears reasonable to assume that in addition to immune cells infected in the tonsil, immune cells present in the airway epithelium can play a major role for BVDV dissemination and systemic spread [5,21,23,25].

Measles virus that resembles BVDV in its lymphotropism has developed a different strategy to overcome the epithelial barrier [36,37,43,53]. It recognizes nectin-4, a basolateral protein, as a receptor and therefore can infect polarized cells only via the basolateral plasma membrane [53]. As MV is released from the apical side, infection of epithelial cells can explain the late stage of MV pathogenesis, when the virus gets back to the airway epithelium and is released into the airway system for spread to other hosts [54]. For entering a new host, MV is assumed to rely on immune cells, e.g. macrophages or dendritic cells, that after infection function as a ferry and carry the infectious agent across the epithelial barrier [55,56]. The results of the present study show that, like MV, BVDV is released from the apical side of polarized epithelial cells. Therefore, BVDV and MV may use a similar strategy to get across the airway epithelium.

Despite the similarity with respect to virus release from the apical side of polarized cells, BVDV differs from MV in its capacity to infect these cells not only via the basolateral domain but also via the apical plasma membrane. The concept described above that apical infection of epithelial cells by BVDV is a means to amplify the amount of infectious virus in the airway and thus to increase the chances of virus spread does not apply to MV infection.

Our results indicate that bovine CD46 is an apical protein and mediates apical infection by BVDV. In humans, isoforms of CD46 have been reported that contain signals in the cytoplasmic tail resulting in endocytotic uptake and/or basolateral transport [57,58]. Amino acid sequences reported for bovine CD46 do not contain the respective signal sequence [59]. Furthermore, CD46 present in human airway epithelial cells has also been reported to be predominantly expressed on the apical plasma membrane [40]. Being an apical protein, bovine CD46 can mediate apical infection but not basolateral infection. The observation that airway epithelial cells can be efficiently infected by BVDV from the basolateral side despite the absence of CD46 at the basolateral domain of the plasma membrane provides evidence for an alternative, CD46 independent entry mechanism. In agreement with our conclusion, it has been reported very recently that BVDV can infect a CD46-knockout cell line [60]. Therefore, future attempts should be directed in elucidating the receptor for basolateral infection. In addition, identification and characterization of the viral and cellular determinants involved in the efficient release of BVDV from the apical membrane represent another highly interesting focus for future work and will further enhance our knowledge on BVDV biology.
